# Assessment of Root Canal Anatomy of Mandibular Permanent Incisors in a Sample of Yemeni Population

**DOI:** 10.1155/ijod/2973236

**Published:** 2025-08-02

**Authors:** Mohammed A. Aldawla, Ahmed A. Madfa

**Affiliations:** ^1^Department of Conservative Dentistry and Endodontics, Faculty of Dentistry, Sana'a University, Sana'a, Yemen; ^2^Department of Restorative Dental Science, College of Dentistry, University of Ha'il, Ha'il, Saudi Arabia

**Keywords:** CBCT, computed tomography, mandibular incisors, root canal morphology, second canals, Yemeni population

## Abstract

**Background:** To investigate the root and canal morphology of mandibular using cone-beam computed tomography (CBCT) in a sample of Yemeni population.

**Methods:** A total of 320 (160 females and 160 males) Yemeni patient's CBCT scans were examined. A total of 1280 mandibular incisors (640 mandibular central incisors and 640 lateral mandibular incisors) were evaluated. The following items were recorded: (1) number of roots, (2) number of canals, (3) root canal type, and (4) bilateral symmetry in terms of root canal number and root canal type. The effect of gender bilateral symmetry on the incidence of root canal morphology was also investigated.

**Results:** All mandibular central incisors examined in this study had one root. On the other hand, 99.7% of lateral mandibular incisors were single-rooted only 0.3% had two roots. The overall prevalence of second root canals in mandibular central and mandibular lateral was 48.9% and 37.9%, respectively. Where males 28.4% and 27.3% had more canals than females 20.3% and 17.8% in both mandibular central and lateral incisors, respectively. Type I canal configuration was the most prevalent in both mandibular incisors, followed by Type III in central and lateral incisors 38.9% and 34.2%, respectively. The prevalence of the other configuration types was as follows: Type II occurred in 7.5% of central and 7.03% of lateral incisors and Type V occurred in 0.8% of central and 1.1% of lateral incisors. Types IV, V, VI, VII, 1–2–3, 1–2–1–3, and 2–1–2–1 were also found in both mandibular incisors with relatively less frequency, and Type VIII was not detected at all. The bilateral symmetry in the number of root canal was 99.3% in mandibular central incisors and 99.6% in mandibular lateral incisors. Furthermore, 97.18% of the mandibular central incisors and 93.75% of mandibular lateral incisors had symmetry in terms of Vertucci's canal configuration. With no significant difference between gender.

**Conclusions:** Mandibular incisors in the Yemeni population are mainly single rooted with 0.3% having two roots. The occurrence of the second canal in central and lateral mandibular incisors is approximately 46.9% (Type I was the predominant followed by Type III). When at least one tooth had two root canals, bilateral symmetry between contralateral teeth was found in 99.3% and 99.6% of cases for central incisors and lateral incisors, respectively. Males have more incidence of second canals than females.

## 1. Background

The efficacy of root canal treatment relies on sufficient chemomechanical preparation and efficient filling of the root canal system. To reach every location in a root canal using mechanical and chemical techniques, a thorough understanding of the anatomy of root canal systems and their variations is a necessary precondition [1]. Anatomical aspects of teeth's roots and root canals should be well understood, as this will reduce the number of root canals missed during treatment and raise the clinical success rate [2]. Insufficient understanding of the many types of root canals can result in a canal being overlooked, leading to ongoing inflammation around the root tip and ultimately causing many root canal treatments to fail [3]. From this perspective, it is particularly important to highlight how a decrease in the success rate of endodontic treatment can also translate into increased mechanical stress on modern rotary instruments. Despite advances in alloy composition, design, and dimensional engineering that have significantly improved the safety and efficacy of endodontic tools, unexpected anatomical challenges—if not properly identified and managed—can impose unforeseen mechanical demands. These added stresses can compromise even the most advanced systems, increasing the likelihood of instrument separation or procedural failure, as demonstrated in the study by Seracchiani et al. [4]. Unfortunately, the root canal system has considerable anatomical variability among different cultures [5], within the same population [6], and even within the same type of tooth. Therefore, having a thorough understanding of the canal root, and canal morphology of a certain population is crucial for creating and performing access cavities that provide direct access to the main root canals.

The mandibular permanent incisors are the earliest permanent teeth to erupt and have a crucial impact on both appearance and speech [7]. Nevertheless, their positioning in the anterior region renders them vulnerable to fractures caused by trauma [8]. The presence of sublingual salivary glands in close proximity enhances the occurrence of perio-endo lesions and dental caries. This is caused by the accumulation of a substantial amount of calculus on the lingual side [8, 9]. These problems may require endodontic therapy; hence it is important to comprehend the root canal anatomy of these teeth. The root canal systems of these teeth with a single root typically consist of a single root canal. However, numerous studies have demonstrated that the root canal structure of mandibular incisors is more complex than it appears on periapical X-rays. These teeth may have additional canals, such as bifurcated or second lingual canals, which further complicate their anatomy. Given this, the integration of three-dimensional imaging, particularly cone-beam computed tomography (CBCT), should be considered when complex root anatomy is suspected. While it is crucial to acknowledge the increased exposure to ionizing radiation, the diagnostic benefits of CBCT in such cases are substantial. CBCT enables a more accurate visualization of root canal systems, thereby improving canal detection rates and treatment planning accuracy. When incorporated into a comprehensive treatment strategy, the use of CBCT is justifiable and aligns with the principle of achieving the highest standard of care. The recent work by Reda et al. [10] underscores this point, demonstrating how CBCT can enhance anatomical classification and guide more precise endodontic interventions. The structure of the mandibular incisor poses difficulty during endodontic access due to its diminutive size and frequent occurrence of two canals. The main cause of failure in root canal therapy of mandibular incisor teeth is the challenge of identifying a second canal, which cannot be properly disinfected and filled during treatment [8, 11].

There is variation in the occurrence of a second canal in mandibular incisor teeth among different populations. Vertucci's study found that the occurrence of a second canal was 25.7% [12]. In the Chinese population, the incidence of a second canal in the mandibular central and lateral incisors was 5.71% and 27.36%, respectively [13]. In the Saudi population, the incidence was 30% [14], while in the north Jordanian population it was 26.2% [15]. In the northeast Indian population, the incidence was 36.25% [16]. The occurrence of two canals in the mandibular central and lateral incisors in the Iranian population was found to be 27.3% and 29.4%, respectively [17]. A study conducted in the Turkish population found the highest occurrence (63%) of a second canal in mandibular incisors [6]. Another significant variable in the root canal morphology of mandibular incisors is the presence of diverse root canal designs, in addition to the number of root canals in a root canal system. The majority of populations have been seen to have Vertucci Type I as the predominant type in their mandibular incisors [5, 13, 18–20]. When a second canal is present, Type III is the predominant root canal configuration in many populations [18, 21–23]. However, some studies have reported that Vertucci Type II is more frequently observed than Type III as the second most common root canal design [15, 17, 24–27].

Several studies have been conducted to determine the morphological characteristics of the root canal system. These studies have utilized various techniques, such as tooth-clearing technique, ground sectioning, three-dimensional wax models, digital radiography, resin injection, radiographic methods with radiopaque contrast media, scanning electron microscopy, stereomicroscope, CBCT, and micro-CT. The clearing approach has been used for a significant period of time [27–37]. However, this technique can be deleterious when used to determine the structure and produces artifacts that may not accurately represent the true morphology of the root canal [38–40]. While micro-CT has successfully addressed these constraints by offering precise and detailed quantitative and qualitative measures of root canal morphology with high precision and resolution [39, 41], it remains inaccessible in undeveloped and emerging nations. Moreover, the expense and level of radiation exposure associated with micro-CT are additional considerations. Furthermore, the CBCT approach has been employed to assess the anatomy of root canals [34]. This instrument offers a suitable solution for doctors to perform noninvasive and three-dimensional reconstruction imaging in endodontic applications and morphological analyses [13, 42, 43]. CBCT has demonstrated its dependability and precision in identifying the quantity and structure of root canals, in contrast to conventional techniques like clearing and micro-CT [39, 44]. Furthermore, CBCT is a readily accessible and cost-effective technique that can be employed either in vivo or ex vivo.

The Yemeni population has a significantly higher occurrence of dental caries compared to other populations, indicating a greater need for root canal therapy among Yemenis [45]. However, there is currently no existing database on the root canal anatomy of permanent mandibular incisors among the Yemeni population. Therefore, it is necessary to conduct research to address this knowledge gap. Hence, the objective of this study was to examine the structure and shape of the roots and canals in mandibular incisors among individuals from Yemen using CBCT imaging.

## 2. Method

### 2.1. Study Design

The protocol of this study, which involved observing and describing a specific population at a certain point in time, was authorized by the Medical Ethics Committee of the University of Sana'a in Yemen. All people who participated in the study provided informed consent.

### 2.2. Study Population

The population of Sana'a city is composed of individuals from many governorates across the country. Thus, they can be regarded as an accurate representation of the ethnic diversity of the Yemeni population. The participants in this study were selected among Yemeni persons who were studying at the dentistry college of Sana'a University, in collaboration with a private dental radiology center situated in the central area of Sana'a city.

1300 CBCT images for mandibular incisors were collected. Patients younger than 18 years old or older than 35 years old, were not included in the study.

The selection of CBCT images was made after reviewing the predetermined inclusion and exclusion criteria. Inclusion criteria included individuals with;

i. Untreated permanent mandibular incisors
ii. No deep dental caries or lesions, root fillings, or fiber post restorations
iii. Fully developed roots
iv. CBCT images of good quality within the permanent mandibular anterior region.

Individuals were excluded from the study if they are as following:

i. Pregnant women
ii. Internal resorption
iii. Canal obliteration
iv. Crown or root fracture
v. Previously treated teeth with restoration or RCT
vi. Missing one of the mandibular incisors
vii. At least one incisor has an immature open apex root

The sample size was calculated using Cochran's formula for estimating sample size equation as follows:

  $$\begin{array}{c} N=\left(Z\alpha \times P\left(1-P\right)\right)/D^{2}, \end{array}$$

where*N*: minimum sample size; α is 0.05 and the critical value is 1.96); *Z*α: the critical value of the normal distribution at α/2 (e.g., for a confidence level of 95%, and *P*: prevalence of the second canal (30%), based on the previous study [32]; and D: degree of precision. The recommended sample size was 320 teeth. Once 1300 images were reviewed for inclusion and exclusion criteria, 320 CBCT scans made up the study's final sample size.

### 2.3. Image Acquisition

The CBCT images evaluated in this study were performed with minimum exposure necessary for adequate image quality. The CBCT images were captured by Pax-i 3D GREEN PHT-60CFO (Vatech, Seoul, South Korea). Operated at 60 kVp and 4 mA, with a field of view (FOV) of 5 cm × 5 cm, 8 cm × 5 cm, 8 cm × 8 cm, exposure time of 9–15 s, and a voxel size of 120 μm.

### 2.4. CBCT Recording

After recording the gender, the subsequent morphological characteristics of the mandibular incisors were examined and recorded by carefully analyzing the teeth from different perspectives: axial, sagittal, and coronal planes. The following data were observed:

• Number of roots
• Number of root canals
• Type of root canals according to Vertucci's classification
• Bilateral symmetry in terms of root canal number and root canal type.

Two examiners assessed 50 randomly selected CBCT images separately, and any disagreement between them was discussed until a consensus was reached. To test the reproducibility, the two observers reexamined 50 randomly selected CBCT images 6 weeks after the first evaluation. The inter-examiner and intra-examiner agreement of 50 CBCT scans was very high with a Kappa coefficient of 0.86 for root numbers and 0.82 for canal numbers.

### 2.5. Statistical Analysis

Data were analyzed using the Statistical Package for the Social Sciences (IBM Co., New York, NY, USA), including frequency distribution and cross-tabulation. The analysis determined the total number of roots and the structure of the root canals. The chi-square and Fisher's exact tests were employed to ascertain the presence of any association between the patient's gender, location of root, and canal morphology. The level of significance was set at α = 5%.

## 3. Results

A total of 320 patients, 160 female and 160 male individuals, aged 18–35 years (average age 30.44 years) were included in this study. From the 320 patients, 1280 mandibular incisors (640 mandibular central incisors and 640 lateral mandibular incisors) were evaluated. All patients had bilateral mandibular central and lateral incisors.

**Figure 1:**
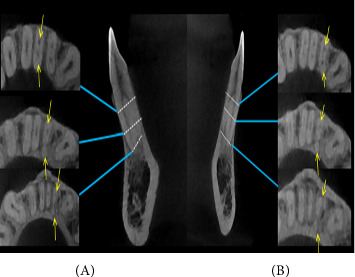
(A, B) CBCT images of two mandibular lateral incisors with two roots. “Dots” represent the axial views of root canal at different levels coronal third, mid-root, and apical third. “Yellow arrow” pointing.

All mandibular central incisors examined in this study had one root. Most lateral incisors were single-rooted (*n* = 638, 99.7%); only 0.3% (*n* = 2, Figure 1) had two roots. Both these lateral incisors were for two men and both on the left side of the arch.

**Figure 2:**
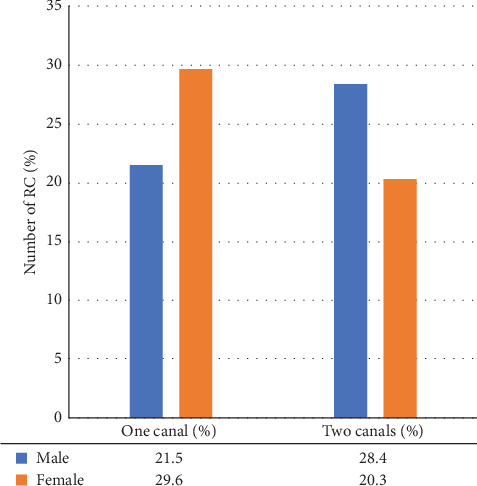
Number of root canal in mandibular central incisors in relation to gender.

**Figure 3:**
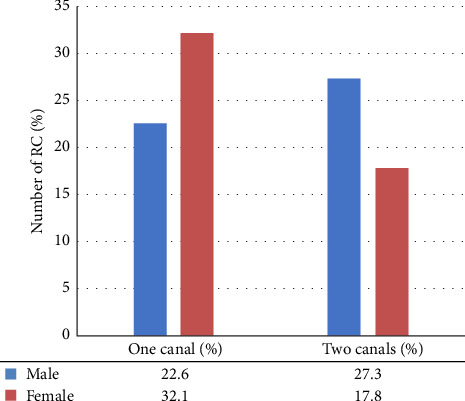
Number of root canal in mandibular lateral incisors in relation to gender.

Most mandibular incisor teeth had one canal. The prevalence of second root canals for mandibular central incisors (*n* = 313, 48.9%) was significantly higher than that for lateral incisors (*n* = 284, 44.3%). For mandibular central incisors, the frequency of second root canals in males (*n* = 182, 28.4%) was higher than in females (*n* = 130, 20.3%) as shown in Figure 2. As well as with mandibular lateral incisors, the frequency of second root canals in male (*n* = 175, 27.3%) was higher than in females (*n* = 114, 17.8%) as illustrated in Figure 3.

**Figure 4:**
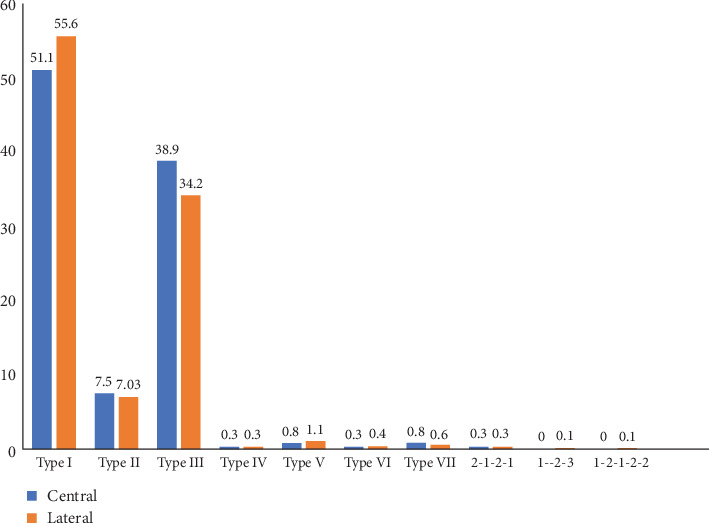
Distribution of root canal types in mandibular incisor teeth.

**Figure 5:**
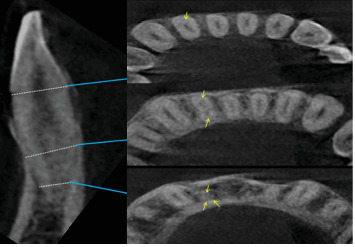
Additional root canal configuration 1–2–3 “dots” represent the axial views of root canal at different levels of coronal third, mid-root, and apical third. “Yellow arrow” pointing to the canals.

**Figure 6:**
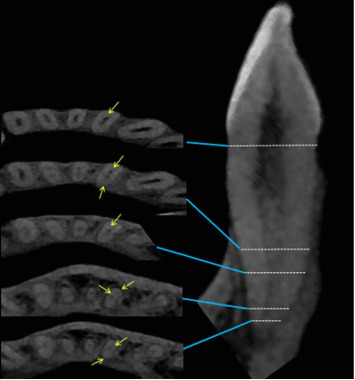
Additional root canal configuration 1–2–1–2–2 “dots” represent the axial views of root canal at different levels of coronal third, mid-root, and apical third. “Yellow arrow” pointing to the canals.

**Figure 7:**
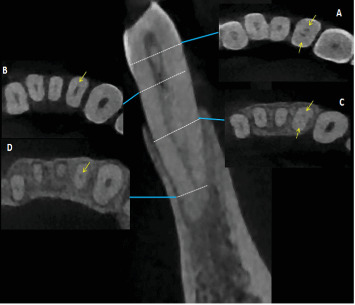
Additional root canal configuration 2–1–2–1 “dots” represent the axial views of root canal at different levels of coronal third, mid-root, and apical third. “Yellow arrow” pointing to the canals.

The frequency of complex root canal configurations in mandibular incisors, irrespective of the gender and the localization of the teeth, revealed that the largest proportion of central (51.1%, *n* = 327) and lateral (55.6%, *n* = 356) incisors had Type I root canal configuration (Figure 4). Type III was the most common root canal Type with two canals [38.9% (*n* = 249) and 34.2% (*n* = 219) in central and lateral incisors, respectively). The prevalence of the other configuration types was as follows: Type II occurred in 7.5% (*n* = 48) of central and 7.03% (*n* = 45) of lateral incisors and Type V occurred in 0.8% (*n* = 5) of central and 1.1% (*n* = 7) of lateral incisors. Types IV, V, VI, and VII were also found in both mandibular incisors with relatively less frequency (Figures 5–7).

Based on the chi-square test results the bilateral symmetry of root canal number and Vertucci's canal configurations were recorded. No significant difference was obtained between left and right sides (*p* > 0, 05). Overall, 99.3% of the central incisors and 99.6% of the lateral incisors had symmetry in the number of canals (Table 1). Additionally, 97.18% of the central incisors and 93.75% of the lateral incisors had symmetry in terms of Vertucci's canal configuration. There was no discernible difference in the root canals between the males and females (*p* > 0, 05).

## 4. Discussion

The exterior appearance of mandibular incisors may not accurately reflect the intricate internal structure of their root canals. For instance, factors such as the arrangement of root canals, the quantity of canals, the presence of accessory canals, communication between canals, the division of canals, and the apical delta. Research investigations have demonstrated that the structure of root canals in mandibular incisors is inconsistent and exhibits significant diversity, both within the same community and when compared to various populations, as observed using Vertucci's configuration. Gender and geography are considered to be factors that contribute to the variance in root canal anatomy. This study is the first to offer a comprehensive analysis of the root canal structure of permanent mandibular incisors in the Yemeni population, utilizing CBCT imaging.

The findings of the present investigation demonstrated that every mandibular central incisor possessed a single root (*n* = 640, 100%). The majority of lateral incisors (99.7%, *n* = 638) were single-rooted, while only a small percentage (0.3%, *n* = 2) had two roots. These data indicate that the presence of mandibular incisors with two or more roots is infrequent. Both of these lateral incisors were located on the left side of the arch and belonged to two different individuals. These findings aligned with the results reported by Zhengyan et al.[46], Monsarrat et al. [19], and Kayaoglu et al. [47], but differed from the majority of other studies where only one root was observed in all mandibular incisors [5, 13, 17, 18, 20, 48–51]. The observed discrepancies can be ascribed to disparities in racial ancestry, genetic components, and methodologies employed in research.

The present study found that second canals were present in 48.9% of the central incisors, 44.3% of the lateral incisors. This result was higher than Vertucci [12], who found the prevalence of second canals in mandibular central incisors was 30%. In Saudi Arabian, Howait et al. [52] found out the prevalence of two canals in mandibular central and lateral incisors was 20.1% and 23.2%, respectively. Furthermore, Han et al. [13] reported that the prevalence of second root canals for mandibular lateral incisors 27.36% was significantly higher than that for central incisors 15.71% in the China population. In Iranian population, Mirhosseini et al. [53] reported the prevalence of second root canal in mandibular lateral teeth 35% was more than the mandibular central teeth 23.9%. Also in the Brazil population, Silva et al. [21] examined 1,200 maxillary and mandibular anterior teeth from patients who required CBCT in the Brazil population. They observed that the proportions of mandibular incisors with two canals were high in mandibular lateral incisors 39.5% compared to mandibular central incisors 35.5%. In Greece populations, Kalaitzoglou et al. [23] found out of 143 central and 146 lateral mandibular incisors the higher incidence of the presence of a second canal was detected in mandibular lateral incisors 30.1% than mandibular central incisors 28.7%. The results of this study were consistent with Arslan et al. [5], who reported that mandibular central incisors 48.1% had two canals more than mandibular lateral incisors 47.1%. The Georgia [20] population Beshkenadze examined mandibular incisors in vivo utilizing CBCT and reported that 45.9% of mandibular central incisors had two canals with less prevalence than 47.1% of mandibular lateral incisors. Such differences in the prevalence of second root canals among mandibular incisors may be attributed to variations in examination techniques, regional, and ethnic distributions of teeth and diversity in the sample size.

Numerous studies have shown that the primary factor responsible for failure of the root canal treatment is the inadequate quality of the chemomechanical disinfection of the root canal system [54, 55]. Chemomechanical preparation of root canal system and 3D obturation is easier in Type I, Type II, and Type IV root canals, as each canal emerges distinctly from the pulp floor and can be located easily with the help of an operative microscope. Teeth with Type III are considered difficult to be instrumented and cleaned as the canal division occurs below the level of the pulpal floor in the radicular area. Most of the mandibular incisors in this study had a uniform pattern, with a single root and a single canal. Al-Qudah and Awawdeh [15] reported that 73.8% of the mandibular incisors of Jordanian population were found to have Type I canal configuration. Caliskan et al. [56] have reported that 68.63% of the mandibular incisors had a Type I canal configuration in the Turkish population. Arslan et al. [5] reported that the Type I canal configuration of the mandibular central and lateral incisors was 51.9% and 52.9%, respectively, in the Turkish population. In the present investigation, Type I root canal configuration was present in 51.1% of mandibular central incisors and 55.6% of mandibular lateral incisors, lower than other studies [13, 18, 19, 57], but in accordance with Arslan et al. [5] and Beshkenadze and Chipashvili [20]. When a second canal is present in this study, Type III was the most common root canal configuration in central and lateral incisors (38.9% and 34.2%), respectively. The prevalence of the other Types of configurations was as follows: Type II occurred in 7.5% of mandibular central incisors and 7.03% of mandibular lateral incisors and Type V occurred in 0.8% of mandibular central incisors and 1.1% of mandibular lateral incisors. Types IV, V, VI, VII, 1–2–3, 1–2–1–3, and 2–1–2–1 were also found in both mandibular incisors with relatively less frequency, and Type VIII was not detected at all. These results are in agreement with a study conducted in the China population by Lin et al. [18], he found that the second most common canal type in mandibular central incisors 6.2% and mandibular lateral incisors 19.3% was Type III. Furthermore, similar findings have been reported in various populations, including Brazil where Nogueira Leal da Silva et al. [21] founded that Type III had a higher prevalence percentage following Type I with 18% in mandibular central incisors and 25.5% in mandibular lateral incisors. The discrepancies in the root canal structure of mandibular incisors found in the literature may be attributed to variances in ethnic background and the methodology employed to analyze the root canal anatomy. In addition, the size of the sample may also have a role.

This study also included the study of sex-specific correlation of second canals, which showed that in mandibular central incisors, the frequency of second root canals in males (*n* = 182, 28.4%) was higher than in females (*n* = 130, 20.3%). As well as with mandibular lateral incisors, the incidence of second root canals in males (*n* = 175, 27.3%) was higher than in females (*n* = 114, 17.8%) with no statistically significant difference. These results are in agreement with Mashyakhy and Gambarini [58] who studied the Saudi population and found that the frequency of second root canals in central and lateral mandibular incisors in males (32.7% and 32.8%) was higher than in females (20.6% and 29.0%), respectively. Furthermore, similar findings have been reported in different populations. In the Iranian population, Altunsoy et al. [59] found that the prevalence of second root canals was higher in males (18.9% and 22.8%) than in females (11.8% and 15.78%) in both central and lateral mandibular incisors, respectively. In addition, Kayaoglu et al. [47] found in Turkish population the higher incidence of second root canals in males (16.2% and 18.8%) than in females (13.4% and 16%) in both central and lateral mandibular incisors, respectively. In contrast to the findings of the above studies, Saati et al. [49] studied the root canal morphology of mandibular incisors conducted in the Iranian population. He reported that the highest prevalence of second root canals in both central and lateral mandibular incisors was in females (15.8% and 21.5%) more than males (15.2% and 21.1%), respectively. In the Turkish population, Sert and Bayirli [6] found that among demographic variables, age, and gender were correlated with two-canal roots. The prevalence of mandibular incisors with two root canals was higher in females than males in their study. Almohaimede et al. [60] studied the root canal morphology of mandibular incisors conducted in the Saudi subpopulation. She found that women had a higher number of mandibular incisors with two canals (322/57.09%) than men (242/42.9%). Difference in study design, differences in evaluation techniques, sample size, and ethnicity may be responsible for the variance in the prevalence of anatomical variation in mandibular incisors among males and females.

Anatomical bilateral symmetry of mandibular incisors in terms of the number of canals and in terms of the canal shape could be clinically useful when using only periapical radiographs for preoperative assessment to treat contralateral incisors in the same patient. This means that if a patient has two canals in a mandibular incisor, the clinician should be aware of the possibility of having a second canal in the contralateral incisor [61]. However, data about bilateral symmetry of root canals in the mandibular incisors in literature contains few studies. Regarding bilateral symmetry for root canal number in mandibular incisors, the present study found results similar to previous studies for mandibular central incisors was 99.3% and for mandibular lateral incisors was 99.6% and when comparing bilateral symmetry of root canal Type between mandibular central and lateral incisors, central incisors 97.18% had a higher percentage of bilateral symmetry than lateral incisors 93.75%. However, there was no significant difference found between sexes in terms of bilateral symmetry. In Saudi Arabia, Howait et al. [52] found out that bilateral symmetry in the number of canals and canal configurations between the right and left mandibular anterior teeth was assessed and found to be present in the majority of cases (96.3%). In the study by Lin et al.[18], which evaluated the symmetry of the number of root canals and Vertucci's canal configuration of permanent mandibular incisors teeth in a Chinese population, 95.2% of the central mandibular incisors and 93.8% of the lateral mandibular incisors had symmetry in the number of canals, and 92.7% of the central mandibular incisors and 89.2% of the lateral mandibular incisors had symmetry in terms of Vertucci's canal configuration. Kayaoglu et al. [47] compared 973 mandibular central incisors and 1010 mandibular lateral incisors to establish bilateral symmetry in root and canal anatomy between left and right sides using CBCT scanning in a Turkish population. They concluded that mandibular central incisors 94.8% exhibited greater symmetry than mandibular lateral incisors 89.8% in the root canal numbers.

There are some limitations that need to be taken into account in the current investigation. The inclusion of data from only one center in the analysis of the current study could be considered a methodological limitation. The forthcoming multicenter research results can more accurately depict the overall population. It is important to take into account the impact of patients' age on the root canal anatomy in future study. While it may limit the generalizability of the findings, we believe the study still provides valuable preliminary data that could inform future, larger-scale research.

## 5. Conclusions

Based on the limitations inherent in the present investigation, it can be concluded that the prevalence of second canals was higher in mandibular central incisors 48.9% than mandibular lateral incisors 44.3%. The Type I root canal architecture was the most prevalent shape observed in both mandibular central and lateral incisors. When a second canal is present, Type III was the predominant root canal configuration in central and lateral incisors. Males exhibited greater morphologic parameters compared to females. The results of our study emphasize the wide range and intricate nature of the root canal structure in the mandibular incisors of individuals from Yemen.

## Figures and Tables

**Table 1:** Bilateral symmetry of root canal number.

Root canal number	Mandibular central incisors			Mandibular lateral incisors		
	Right	Left	Number of symmetrical teeth	Right	Left	Number of symmetrical teeth
One root canal *n*	163	165	163	176	175	175
Two root canals *n*	157	155	155	144	145	144
Total symmetry *n* (%)	—	—	318 (99.3%)	—	—	319 (99.6%)

## Data Availability

The data are available upon request due to privacy/ethical restrictions.
